# Heartbeat: a multimodal dataset of fetal echocardiography and clinical metadata for early detection of congenital heart disease

**DOI:** 10.3389/fcvm.2026.1726484

**Published:** 2026-04-15

**Authors:** Santiago Rodríguez, Alejandra Pérez, Lina Marcela Echeverry, Ángela Castillo, Nataly Alejandra Ramírez, María Escobar, Sofía Guarín Monroy, Daniela Vega, Nicolás Rodríguez, Camila Castro-Páez, Javier Navarro, María Teresa Domínguez, Nicolás Laverde, Luis Andrés Sarmiento, Daniel Afanador, Liz D'silva Londoño, Erika Torres Narváez, María Juliana Fandiño, Antonio José Madrid, Juan Carlos Quintero, Nadiezhda Rodríguez, Juan Carlos Briceño, Pablo Arbeláez

**Affiliations:** 1 Center for Research and Formation in Artificial Intelligence (CinfonIA), Universidad de los Andes, Bogotá, Colombia; 2 Facultad de Medicina, Universidad El Bosque, Bogotá, Colombia; 3 Facultad de Medicina, Universidad de los Andes, Bogotá, Colombia; 4 Escuela de Graduados, Doctorado en Epidemiología, Universidad CES, Medellín, Colombia; 5 Departamento de Ingeniería Biomédica, Universidad de los Andes, Bogotá, Colombia; 6 Departamento de Ingeniería Industrial, Pontificia Universidad Javeriana, Bogotá, Colombia; 7 Departamento de Investigaciones, Fundación Cardioinfantil – Instituto de Cardiología, Bogotá, Colombia; 8 Facultad de Ciencias de la Salud, Pontificia Universidad Javeriana, Cali, Colombia; 9 Departamento de Ginecología, Obstetricia y Reproducción Humana, Hospital Universitario Fundación Santa Fe de Bogotá, Bogotá, Colombia; 10 Clínica Imbanaco, Cali, Colombia; 11 Hospital Universitario del Valle, Cali, Colombia

**Keywords:** artificial intelligence, congenital heart disease, echocardiography, multimodal data, prenatal detection, transformers

## Abstract

**Background:**

Congenital heart diseases (CHDs) remain the leading cause of infant mortality attributable to birth defects. Although artificial intelligence has demonstrated promise for automated CHD detection from prenatal ultrasound imaging, progress is constrained by the limited availability of large, high-quality datasets and the absence of multimodal resources integrating imaging with clinically relevant patient metadata.

**Methods:**

This study introduces Heartbeat, a multicenter, anonymized multimodal dataset comprising fetal cardiac ultrasound images and associated clinical metadata collected between 2019 and 2023. Standard echocardiographic views, including the three-vessel trachea, four-chamber, left ventricular outflow tract, and right ventricular outflow tract planes, were acquired according to established protocols. Multiple deep learning architectures, including convolutional neural networks and a transformer-based baseline, were trained and evaluated. Heart-ViT, a multimodal transformer-based model, was developed to integrate imaging features with patient-specific clinical metadata via adaptive layer normalization.

**Results:**

Heartbeat includes 1,475 patients, with CHD prevalences of 6.50% and 7.25% in the second and third trimesters, respectively. On the second-trimester test set (n=61), Heart-ViT achieved a sensitivity of 79.17%, specificity of 93.18%, PPV of 56.95%, AUROC of 88.56%, and an F1-score of 65.63%, outperforming multiple image-only convolutional neural network architectures and baseline models. Multimodal integration yielded a 13.22-point improvement in F1-score relative to the imaging-only transformer baseline.

**Conclusion:**

Heartbeat provides a clinically relevant multimodal resource for prenatal CHD research that reflects real-world imaging variability and class imbalance. Integrating ultrasound imaging with patient-specific clinical metadata significantly enhances CHD detection compared with image-only approaches. These findings establish a reproducible benchmark and support the development of AI-assisted tools for early prenatal screening. The dataset and analytical framework will be made publicly available to support further research in prenatal cardiology and the early detection of CHDs.

## 1 Introduction

Congenital heart diseases (CHDs) are structural abnormalities of the heart or great vessels that are present from birth (1). Clinical diagnosis of CHDs primarily relies on fetal echocardiography, which demands expert interpretation of intricate cardiac structures visualized by ultrasound (2–4). Despite significant advances in fetal imaging, CHDs remain a leading cause of neonatal morbidity and mortality (5–7), affecting approximately 1% of live births worldwide (8).

Early and accurate prenatal detection of CHDs is essential, as it enables timely intervention and coordinated care, allowing families and clinicians to plan delivery at specialized centers equipped for neonatal cardiac management (9). Such preparation has been shown to significantly improve survival rates and long-term outcomes (10–12). Artificial intelligence (AI) has demonstrated significant potential in medical imaging analysis, contributing to improvements in efficiency, reproducibility, and diagnostic accuracy across various clinical applications (13). In the context of CHDs, AI-driven approaches have been developed to identify cardiac abnormalities in fetal echocardiograms (14, 15) and pediatric chest x-ray images (16).

However, progress in this area remains limited by three major challenges: the absence of public datasets, the lack of multimodal data, and the scarcity of CHDs-specific AI models. On the one hand, most previous studies rely on private institutional datasets (17–20), which limits reproducibility and slows progress toward the development of automated diagnostic tools. On the other hand, although clinical assessment of fetal cardiac health relies on both imaging data and additional clinical factors (such as gestational age, maternal characteristics, and fetal growth parameters) (21–24), existing datasets for prenatal CHDs diagnosis are restricted to ultrasound imaging alone. This lack of multimodal resources forces AI models to rely solely on imaging data, hindering the development of robust approaches that can integrate complementary clinical information to contextualize echocardiographic findings and improve diagnostic accuracy. Finally, while standard deep learning architectures are publicly available, domain-specific CHDs models rarely release their code, further constraining research in this field.

To address these limitations and advance research in this area, this study introduces Heartbeat, a curated and anonymized multimodal database that integrates fetal heart ultrasound images with patient-specific clinical metadata. This dataset is designed to foster reproducible and data-driven research in prenatal CHDs diagnosis by providing a foundation for developing and benchmarking multimodal AI models that more closely reflect clinical practice, where imaging and patient information are combined to support more accurate diagnoses. To demonstrate the potential of the dataset, different deep learning models are trained and evaluated. Moreover, this paper proposes Heart-ViT, a transformer-based architecture that jointly analyzes imaging and clinical data for the early detection of CHDs. This framework aims to evaluate the utility of multimodal integration and to determine whether incorporating patient-specific clinical metadata with echocardiographic images improves detection performance compared with image-only approaches. To facilitate reproducibility and support future research, the Heartbeat dataset and the source code of Heart-ViT are publicly available at https://github.com/BCV-Uniandes/Heartbeat.git.

## 2 Materials and methods

### 2.1 Heartbeat

#### 2.1.1 Dataset collection

The dataset was developed in collaboration with three major healthcare institutions in Latin America: Fundación Cardioinfantil (Bogotá, Colombia), Fundación Santa Fe de Bogotá (Bogotá, Colombia), and Clínica Imbanaco (Cali, Colombia). These institutions are tertiary referral centers specializing in maternal–fetal medicine and fetal cardiology, where patients are frequently referred for evaluation of high-risk pregnancies and suspected congenital anomalies. Data were collected from retrospective exams of more than 1,400 patients with and without CHD spanning from 2019 to 2023.

To protect patient identities, strict anonymization protocols were followed. The images used in this study were stored on the REDCap platform, a widely recognized and internationally trusted system that provides robust data protection. This platform ensures that sensitive information is securely managed in accordance with ethical and legal standards for safeguarding patient privacy. Each participating institution’s ethics committee rigorously reviewed and approved the project, establishing a strong ethical foundation for data use.

#### 2.1.2 Data description

Each patient record consists of both echocardiographic and clinical data, with some patients having multiple exams conducted at different points in time, allowing for insights into the progression of the pregnancy.

#### 2.1.3 Visual data

2D images were acquired using Voluson E6, E8, and E10 systems (GE Healthcare, Austria), following established protocols for prenatal echocardiographic imaging (25). For each examination, a specialist obtained images from standard echocardiographic view planes, which capture different anatomical perspectives of the fetal heart. Specifically, images were collected from the three-vessel trachea (3VT), four-chamber (4C), left ventricular outflow tract (LVOT), and right ventricular outflow tract (RVOT) views. The inclusion of multiple perspectives facilitates the identification of CHDs markers across varying angles and anatomical orientations.

#### 2.1.4 Clinical metadata

Metadata known to influence fetal cardiac development and the diagnostic accuracy of prenatal imaging were collected. These included gestational age, due to its impact on cardiac maturation of CHDs detection during ultrasound evaluation (21); maternal age, which has been identified as a potential risk factor for CHDs (22); the number of umbilical cord vessels, since the presence of a single umbilical artery is associated with an increased prevalence of CHDs (23); and fetal growth percentile, as intrauterine growth restriction has been linked to a higher risk of CHDs and altered cardiovascular development (24).

#### 2.1.5 Task definition

To evaluate the proposed model and explore the benefits of multimodal integration, the dataset task was defined as the prenatal detection of CHDs using ultrasound images and patient-specific clinical metadata. This formulation allows the model to jointly leverage complementary information from both modalities, mirroring clinical diagnostic reasoning. For imaging-only models, no clinical metadata was included. Recognizing the inherent class imbalance between CHDs and non-CHDs cases, the task was approached as a detection problem rather than a conventional classification task, ensuring that evaluation metrics emphasize the accurate identification of CHDs cases, the primary clinical objective. To align with real-world diagnostic practice, predictions were aggregated across all available images per patient, with image-level confidence scores averaged to produce a unified, patient-level prediction at inference.

### 2.2 Method

#### 2.2.1 Model overview

**Figure 1:**
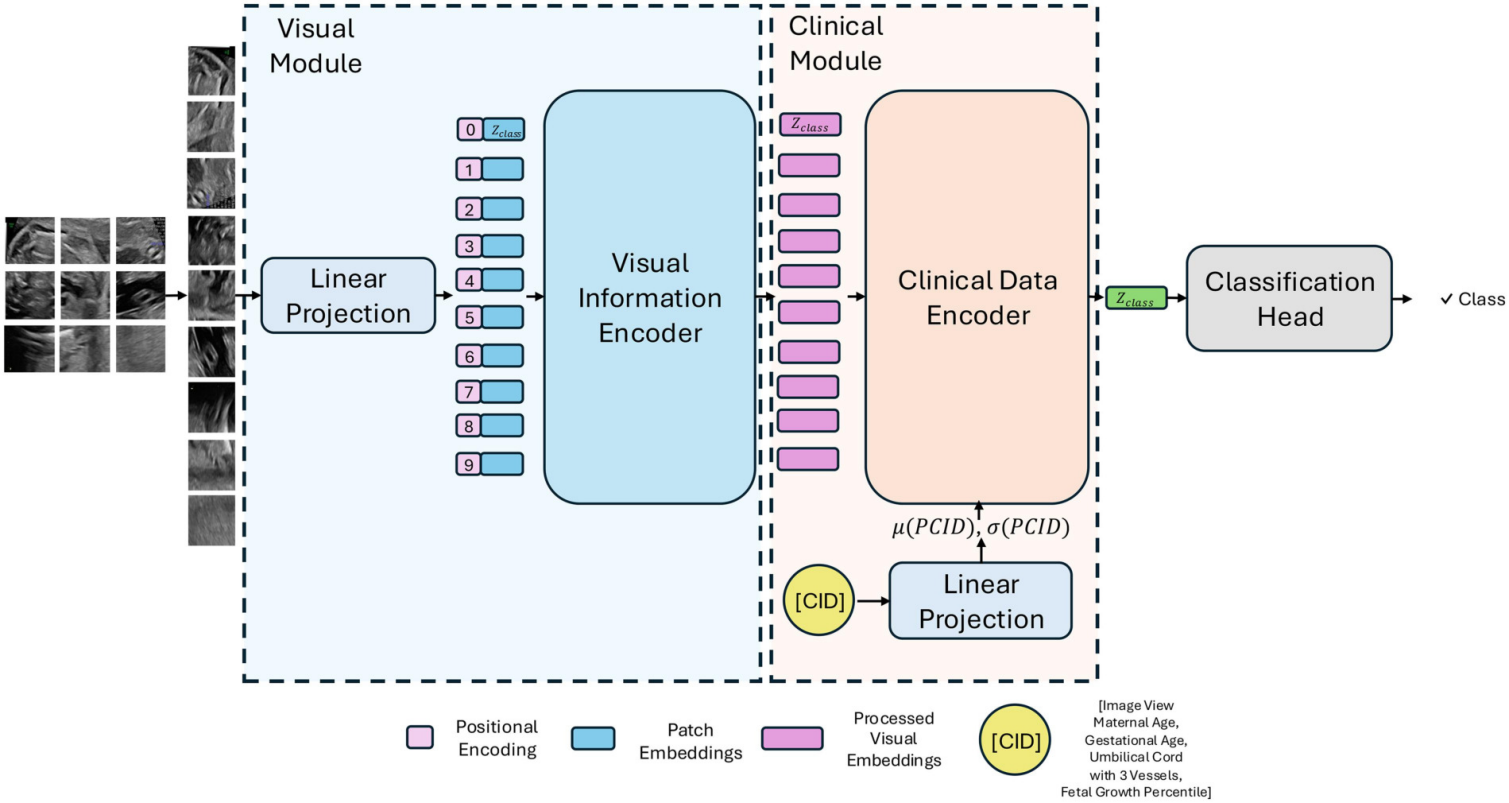
Heart-ViT overview. The architecture consists of a Visual Module and a Clinical Module. The Visual Module divides the input image into 16×16 non overlapping patches, with each patch being linearly projected as an input to transformer encoder layers, which are neural network layers specialized for analyzing sequential data and capturing spatial features. The Clinical Module then takes the visual features from the Visual Module and enriches them with a patient-specific Clinical Information Descriptor (CID), which encodes the patient’s metadata and the imaging view. Transformer encoder layers equipped with AdaIN adjust the visual representations using the mean (μ) and standard deviation (σ) of the linearly CID, allowing the model to adapt image features based on clinical context. The final prediction is made using the class token $Z_{\mathrm{class}}$, a dedicated representation that summarizes classification information from the Clinical Module output.

Heart-ViT is a transformer-based multimodal model designed to integrate imaging and clinical information for the early diagnosis of CHD. As shown in Figure 1, this model consists of two modules: a Visual Module for imaging data and a Clinical Module for patient-specific information. These modules are combined to provide personalized, context-aware predictions, mirroring how clinicians interpret both imaging and clinical context when making diagnostic decisions.

#### 2.2.2 Visual module

The Visual Module is based on the Vision Transformer (ViT) architecture (26), which divides each input image into 16×16 small non-overlapping patches and processes them as a sequence. Using transformer encoder layers with multi-head self-attention, the model learns both local and global spatial relationships across the image. For classification, a dedicated class token summarizes the information from all patches and is passed forward as the image-level representation.

#### 2.2.3 Clinical module

To integrate image and patient information, a Clinical Information Descriptor (CID) is constructed. The CID incorporates the imaging view, specifying which of the four standard planes described in Section 2.1.3 the image was acquired from, as well as the clinically relevant variables for CHD diagnosis described in Section 2.1.4. The Clinical Module processes this CID using Transformer layers with Adaptive Instance Normalization (AdaIN) (27). Unlike conventional normalization, AdaIN conditions the image feature representations on the patient’s clinical profile, effectively adapting feature extraction to each individual case. This design enables the model to incorporate both imaging findings and patient-specific context when generating predictions. The final classification is derived from the Clinical Module’s class token, which combines visual and clinical information.

#### 2.2.4 Evaluation metrics

Previous studies have often employed classification metrics such as accuracy to evaluate overall model performance across all images (19, 28, 29). However, these metrics do not specifically address the model’s ability to identify the positive class and instead provide only a general assessment of classification capabilities. In this study, model performance was primarily evaluated using the F1-score for CHD detection, as this metric balances Positive Predictive Value (PPV) and sensitivity through the harmonic mean. This balance is particularly important in imbalanced clinical settings, where minimizing missed CHD cases and limiting excessive false positives are essential. To further enhance clinical interpretability, additional metrics, including sensitivity, specificity, PPV, and the area under the receiver operating characteristic curve (AUROC), were also computed on the second-trimester test set. This choice ensured that all clinically relevant evaluations were performed on previously unseen data, thereby avoiding bias introduced by the validation set used during model development.

#### 2.2.5 Implementation details

Model optimization was performed using a 4-fold cross-validation scheme on data from the second and third trimesters. Since Heart-ViT method primarily focuses on the second trimester, a dedicated test set was constructed for this period to evaluate generalization performance. The best model trained on the four folds of the second-trimester data was then assessed on this test set. For patient-level prediction, an image-level classification framework was employed, whereby each image was supervised individually to enable the model to learn from single instances. During inference, images were grouped by patient, and predictions were aggregated by averaging the confidence scores across images. The cross-validation set was then used to determine the optimal confidence threshold that maximized the F1-score. This threshold defined the minimum average confidence score required to classify a patient as positive.

For preprocessing, the top 65 pixels of each frame were first removed to eliminate machine-generated metadata and clinical overlays, thereby ensuring patient anonymity. The resulting images were then resized to a standardized resolution of 224×224 pixels and normalized using ImageNet statistics, consistent with common transfer learning practices for Convolutional Neural Networks (CNNs) and ViT architectures pre-trained on ImageNet. During training, data augmentation was applied exclusively to the training set to increase variability and enhance robustness to acquisition-related differences. Random horizontal flips were used to account for left–right orientation variability, random rotations of $\pm 10^{\circ }$ were applied to simulate minor probe angle differences, and random resized crops were performed to improve tolerance to variations in zoom, centering, and field of view. All augmentation procedures were applied consistently across the evaluated architectures.

To address the substantial class imbalance in the dataset, a weighted random sampler was employed, selecting training data points with probabilities inversely proportional to their class frequencies. This sampling method ensured an approximately equal number of positive and negative samples were processed, facilitating more effective learning. Each class was assigned a weight proportional to the inverse of its total sample count, with more common classes receiving lower weights and being sampled less frequently. In addition, a class-balanced loss function (30) with a hyperparameter β of 0.9999 was incorporated to compensate for class imbalance by assigning higher penalties to misclassifications of the underrepresented CHD class. Training was conducted on a Quadro RTX 8,000 GPU with a batch size of 42, using the RAdam optimizer (31) for 40 epochs.

## 3 Results

### 3.1 Dataset summary

**Figure 2:**
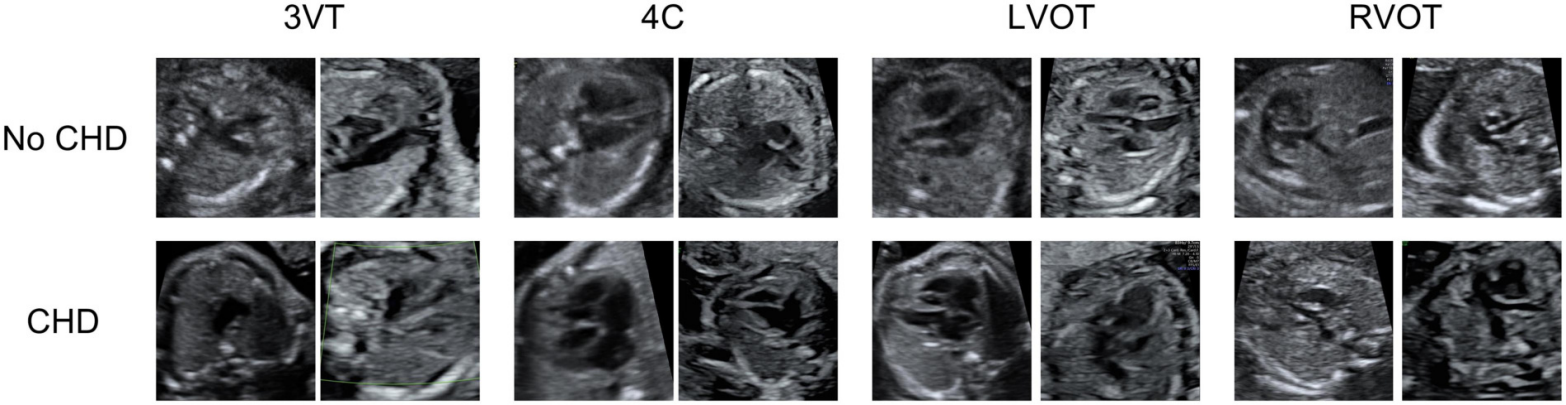
Examples of echocardiographic views from Heartbeat. Representative images of Congenital Heart Disease (CHD) and non-CHD cases are shown for the three-vessel trachea (3VT), four-chamber (4C), left ventricular outflow tract (LVOT), and right ventricular outflow tract (RVOT) views. Each view offers complementary anatomical information that is essential for accurate CHD detection.

**Figure 3:**
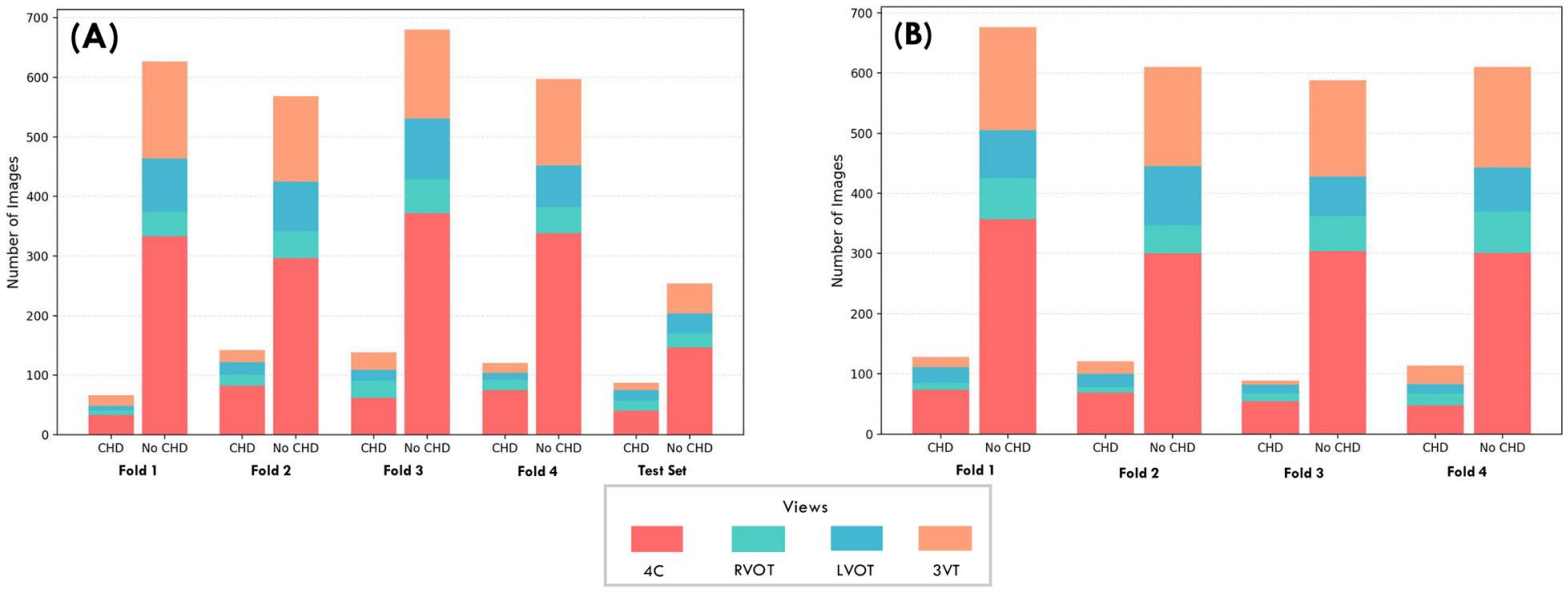
Heartbeat distribution of echocardiography views across data trimesters and folds. The bar charts illustrate the distribution of fetal echocardiography images across the cross-validation setup for **(A)** the Second Trimester dataset and **(B)** the Third Trimester dataset. Each bar details the proportion of specific anatomical views within that fold, including 4C (four-chamber), RVOT (right ventricular outflow tract), LVOT (left ventricular outflow tract), and 3VT (three-vessel trachea).

**Figure 4:**
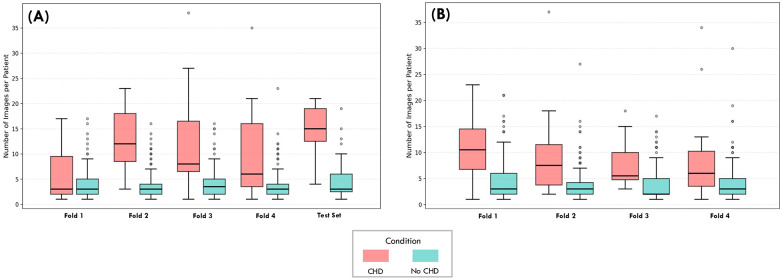
Distribution of images per patient in Heartbeat. The boxplot illustrates the total number of echocardiogram images captured per patient for **(A)** the Second Trimester and **(B)** the Third Trimester datasets.

Heartbeat includes 1,475 patients, with CHD prevalences of 6.50% and 7.25% for the second and third trimesters, respectively. A detailed breakdown of CHDs prevalence across trimesters is presented in Table 1, and representative examples of echocardiographic views are shown in Figure 2. Building on this overview, Figure 3 shows the distribution of images and views, while Figure 4 illustrates the number of images per patient across folds. Most patients diagnosed with CHDs have between 4 and 24 images, whereas patients classified as non-CHD typically have between 4 and 13. Additionally, Table 2 reports the distribution of clinical metadata variables across both groups for each trimester.

**Table 1:** Patient distribution per fold and trimester in *Heartbeat*.

Trimester	Condition	Fold 1	Fold 2	Fold 3	Fold 4	Test	Total (%)
Second	No CHD	169	169	170	171	55	734 (93.50%)
	CHD	12	11	11	11	6	51 (6.50%)
Third	No CHD	160	160	161	159	–	640 (92.75%)
	CHD	12	12	12	14	–	50 (7.25%)

The total reflects the proportion of Congenital Heart Disease (CHD) patients in each trimester.

**Table 2:** Dataset metadata statistics.

Dataset set	Group	Maternal age (years)	Gestational age (weeks)	Fetal growth percentile	Single umbilical artery patients (%)
Second trimester train	CHD	34.40±4.60	22.14±3.08	40.75±23.92	4.44%
	No CHD	34.76±4.81	21.42±3.41	48.64±23.93	0.44%
Second trimester test	CHD	32.56±3.30	22.34±2.37	60.22±21.81	0%
	No CHD	35.24±5.47	21.44±3.58	47.68±21.82	0%
Third trimester train	CHD	34.49±4.91	32.62±3.36	38.35±21.02	4%
	No CHD	35.21±4.68	33.68±3.26	38.03±21.59	0.6%

Baseline maternal and fetal characteristics in the training and test sets for the second and third trimesters, stratified by the presence of Congenital Heart Disease (CHD). Continuous variables are reported as mean ± standard deviation, and categorical variables as counts with percentages relative to the total number of patients within each group.

### 3.2 Model performance evaluation

**Figure 5:**
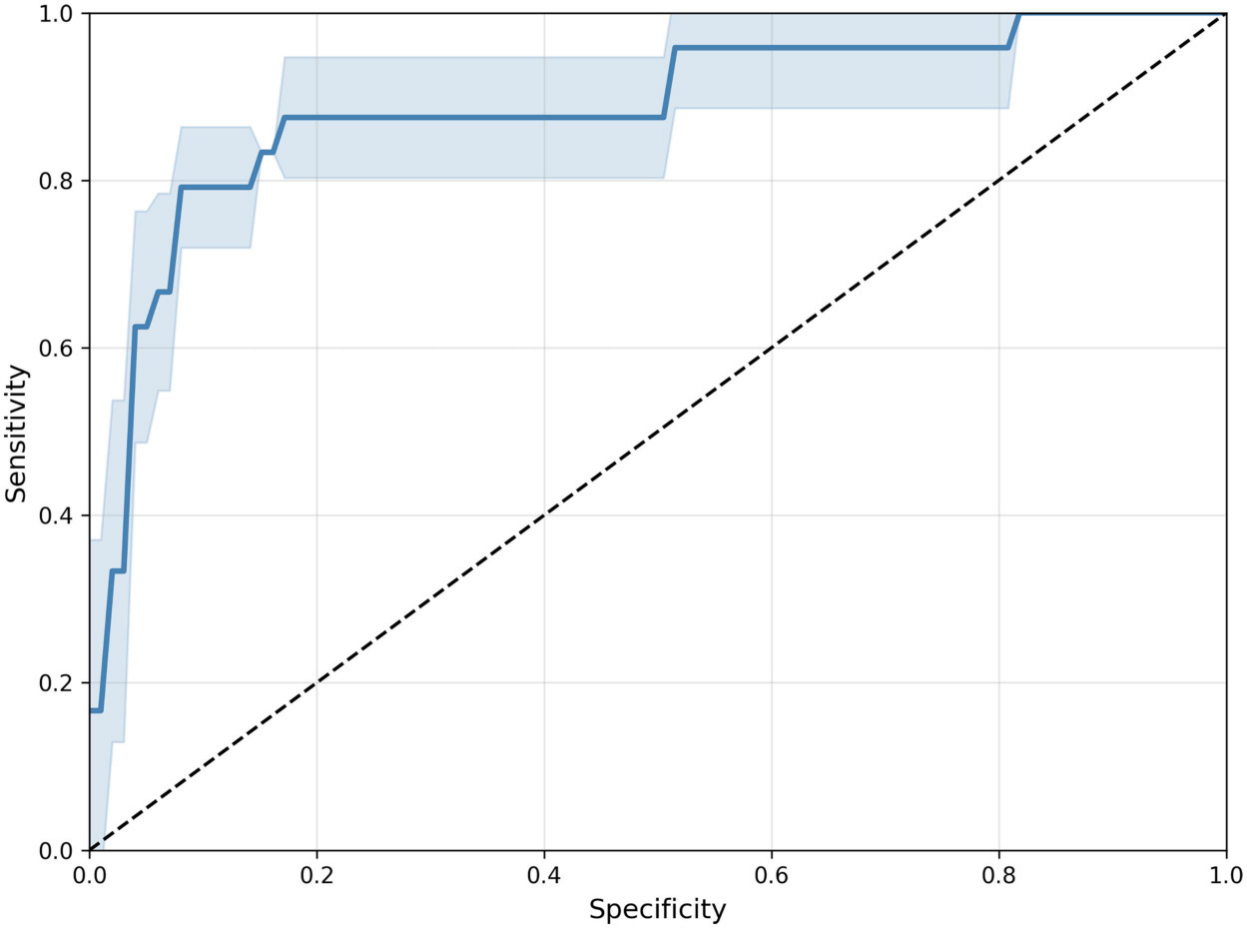
Heart-ViT ROC curve. The solid line represents the mean ROC curve on the test set using the models from each fold, and the shaded area indicates the standard deviation.

**Figure 6:**
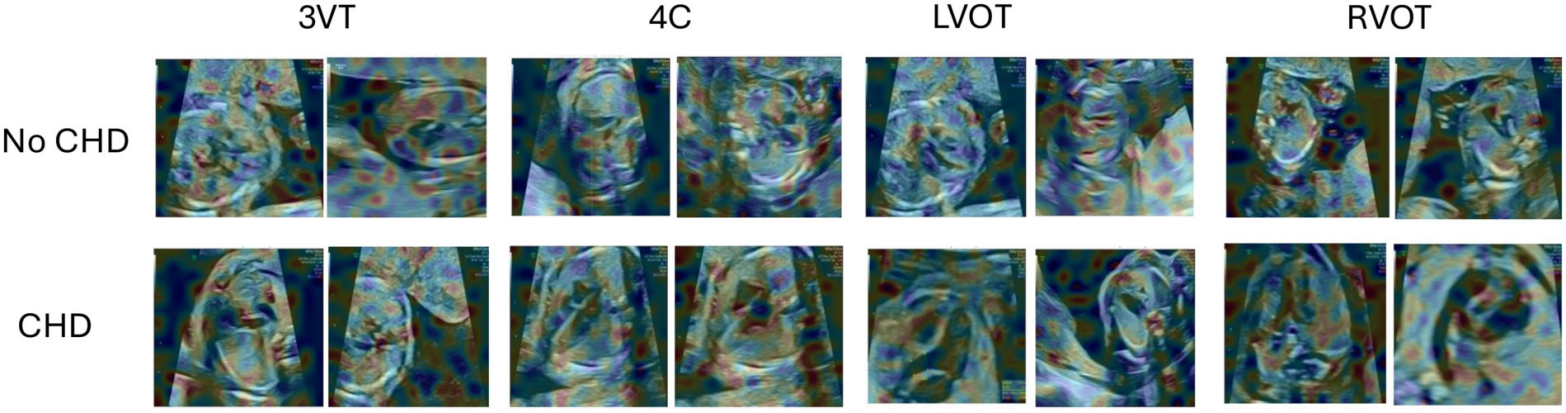
Visualization of Heart-ViT’s attention maps. Attention maps for Congenital Heart Disease (CHD) and non-CHD cases across multiple echocardiographic views. The maps are displayed as heatmaps overlaid on the original images, highlighting the regions of the images that most strongly influenced the model’s predictions.

Table 3 summarizes the performance of all evaluated models on the second-trimester test set. Among CNN-based architectures, ResNet50 achieved the highest F1-score of 49.22±6.23. The transformer-based ViT baseline surpassed all CNNs with an F1-score of 52.40±7.97, representing a 3.19 point improvement over the best-performing CNN. The proposed multimodal Heart-ViT achieved the best overall results, with a sensitivity of 79.17±8.33, specificity of 93.18±2.29, PPV of 56.95±6.99, AUROC of 88.56±3.80, and F1-score of 65.63±2.09, corresponding to a 13.22 point improvement over the ViT baseline. Figure 5 shows the mean ROC curve of Heart-ViT in the test set, demonstrating strong discriminative ability across thresholds, with minimal performance overlap among folds. Moreover, Figure 6 illustrates the mean attention maps from the last transformer encoder layer, which gives insight into Heart-ViT’s decision-making process. These maps illustrate the regions of the echocardiographic images that contributed most to the model’s predictions for CHDs and non-CHDs cases.

**Table 3:** Comparison of state-of-the-art methods for classifying congenital heart diseases (CHDs) on the second-trimester dataset’s test set.

Model	Sensitivity	Specificity	Positive predictive value	AUROC	F1-CHD
MobileNet v2 (32)	29.17±25.00	87.73±7.62	15.30±11.87	82.73±5.06	19.90±15.98
VGG16 (33)	62.50±15.96	88.64±6.86	41.28±12.77	85.46±2.98	47.48±5.02
ResNet18 (34)	66.67±23.57	87.73±4.78	37.30±4.89	86.44±4.32	46.67±9.03
ResNet50 (34)	75.00±9.62	85.46±4.92	37.45±8.60	85.53±2.48	49.22±6.23
ViT-Base (Baseline) (26)	70.84±8.33	88.64±5.01	42.70±11.13	85.83±2.73	52.40±7.97
**Heart-ViT (Ours)**	79.17±8.33	93.18±2.29	56.95±6.99	88.56±3.80	65.63±2.09

Results are reported as mean ± standard deviation across folds. The highest-performing results are highlighted in bold.

A view-wise performance analysis using Heart-ViT was conducted to compare the performance of individual echocardiographic views for CHD detection. As shown in Table 4, the LVOT view achieved the highest F1-score of 70.13±8.13 and PPV of 89.19±3.67, while the RVOT view demonstrated the highest sensitivity of 67.65±24.07. In contrast, the 3VT and 4C views yielded lower F1-scores.

**Table 4:** Heart-ViT view-wise performance comparison.

View	Sensitivity	Positive predictive value	F1-CHD
3VT	43.75±20.73	72.49±9.32	51.99±15.92
4C	51.25±11.79	58.57±5.43	54.19±8.65
RVOT	67.65±24.07	66.87±10.90	66.03±17.52
LVOT	58.33±10.02	89.19±3.67	70.13±8.13

Results are reported as mean ± standard deviation across cross-validation folds for each echocardiographic view on the second-trimester dataset test set. The highest value for each metric is highlighted in bold.

### 3.3 Performance comparison in the second and third trimesters

Although this study primarily focused on second-trimester prediction, experiments on third-trimester images demonstrated improved cross-validation performance. Heart-ViT achieved an F1-score of 68.00±2.66, representing an absolute improvement of 5.43 points over the second-trimester result of 62.57±8.37.

### 3.4 Ablation experiments

Ablation experiments were conducted on the second-trimester cross-validation set to evaluate the contribution of each component of the proposed method. Table 5 summarizes the contribution of different Heart-ViT’s components. Applying an optimal prediction threshold significantly improved performance compared with the default setting. weighted random sampling further enhanced results by balancing positive and negative cases during training, and weighted loss strengthened the model’s ability to recognize the underrepresented class. Incorporating patient information also improved predictive accuracy, as integrating clinical data into visual features through the CID using AdaIN provided an additional performance boost.

**Table 5:** Heart-ViT ablation studies.

Optimal threshold	Weighted sampler	Weighted loss	Clinical metadata	F1-CHD
	✓	✓	✓	37.86±17.67
✓		✓	✓	54.68±2.15
✓	✓		✓	56.45±10.96
✓	✓	✓		47.89±2.88
✓	✓	✓	✓	62.57±8.37

Experiments assess the impact of removing individual components and training optimization techniques from our model on the second-trimester cross-validation set. A ✓indicates the presence of a specific component. The best results are highlighted in bold.

Table 6 presents the ablation study assessing the contribution of each component within the CID. The results show that the model benefits most from the inclusion of fetal growth percentile information, with its removal resulting in a 9.61-point decrease in F1-score. This was followed by the presence of a single umbilical artery (7.59 point decrease), gestational age (6.11 point decrease), and maternal age (4.63 point decrease). Additionally, excluding the image-view information resulted in a 5.63-point drop in performance.

**Table 6:** Clinical information descriptor (CID) ablation studies.

Model	F1-CHD	Δ F1-CHD
Full CID	62.57±8.36	–
– Fetal Growth Percentile	52.96±6.32	↓9.61±10.49
– Single Umbilical Artery	54.98±4.80	↓7.59±9.65
– Gestational Age	56.46±4.78	↓6.11±9.61
– Maternal Age	57.94±4.73	↓4.63±9.64
– Image View	56.94±5.16	↓5.63±9.83

Each experiment evaluates the impact of individual components within the CID on the second-trimester cross-validation set. To assess the contribution of each element, patient information variables were removed one at a time, along with a separate experiment excluding the image view. The ΔF1-CHD column indicates the decrease in F1-score, reflecting each feature’s relative contribution to model performance. Results for patient metadata are presented in descending order of impact, followed by the evaluation of image view inclusion.

## 4 Discussion

Heartbeat offers a valuable resource for developing deep learning algorithms for early CHD detection. As the first multimodal dataset that combines fetal ultrasound images with patient-specific clinical metadata, it captures complex CHD patterns and provides a foundation for integrated analysis. The observed CHD prevalence in our cohort exceeds the approximately 1% reported in the general population (8). This difference likely reflects the referral nature of the study population, as tertiary fetal cardiology centers frequently evaluate pregnancies with suspected anomalies or elevated clinical risk. Consequently, the dataset represents a referral population, which should be considered when interpreting prevalence-sensitive performance metrics such as PPV.

In terms of imaging, the distribution of echocardiographic views demonstrates appropriate representation of standard planes, including the 4C and 3VT views, consistent with their widespread use in fetal cardiac screening (2). Furthermore, variability in the number of images per patient reflects heterogeneous acquisition patterns that closely mirror real-world clinical practice.

Regarding the patient metadata, maternal age averages approximately 34 years across all sets, with low variability and comparable values between CHD and non-CHD groups. As expected, the mean gestational age corresponds appropriately to each trimester within the dataset. Fetal growth percentile values are also consistent across sets but showed considerable inter-patient variability, as reflected by their large standard deviations. Finally, a slightly higher prevalence of single umbilical artery is observed in CHD cases compared to non-CHD controls, consistent with previous reports associating this condition with an increased risk of congenital heart defects (23).

Results in the second trimester show that transformer-based models, such as ViTs, outperform CNNs in CHD classification. This advantage stems from Vision Transformers’ ability to model long-range spatial dependencies via global self-attention mechanisms [26]. In fetal echocardiography, diagnosis frequently depends on spatial relationships between anatomically distant structures, such as ventricular alignment and outflow tract continuity [3], which may be better captured by architectures that incorporate global contextual reasoning. In contrast, CNNs primarily emphasize localized feature extraction. Furthermore, the observed 13.22-point increase in F1-score for Heart-ViT compared to the ViT baseline highlights the benefit of multimodal integration, as conditioning visual features on patient-specific variables provides complementary diagnostic context.

Heart-ViT also achieves high sensitivity and specificity, indicating a strong ability to identify both affected and unaffected cases correctly. The PPV indicates that some false positives remain, likely due to subtle or overlapping imaging features between CHDs and normal cases. Nonetheless, a moderate PPV is expected in this context, as the primary goal of the proposed model is to maximize sensitivity and ensure that potential CHDs cases are not overlooked during screening. The high AUROC further supports the model’s robustness across different decision thresholds, and the ROC curve lies well above the diagonal, confirming that the model performs substantially better than random classification. In addition, view-wise analysis showed that outflow tract views (LVOT and RVOT) achieved higher performance than the 3VT and 4C views, suggesting strong discriminative capacity for detecting abnormalities of the ventricular outflow structures.

The attention maps show that the model’s focus was distributed across multiple regions of the image rather than confined to a single area, suggesting that Heart-ViT integrates information from various parts of the anatomical structures present in the image when forming its predictions. Although regions of increased attention frequently correspond to clinically relevant landmarks, attention remains broadly distributed across the image and is not consistently concentrated in a single region. This pattern suggests that the model leverages distributed spatial information rather than replicating a stepwise, region-specific diagnostic strategy.

Stronger performance was observed on third-trimester images, likely reflecting improved visualization as structural anomalies become more distinct later in gestation (35). These findings highlight the influence of anatomical maturation on model accuracy. However, the second trimester remains the optimal window for routine cardiac screening. During this period, cardiac structures are sufficiently developed to allow systematic evaluation through standard echocardiographic screening views, while still providing a critical time frame for genetic counseling, clinical referral, and perinatal management planning. While third-trimester detection remains important for identifying late-emerging lesions and coordinating tertiary referrals, second-trimester screening is prioritized because it enables timely intervention and reduces the need for repeat evaluations commonly required at earlier gestational stages (3).

In addition, ablation experiments demonstrate that strategies to address class imbalance, such as weighted sampling and weighted loss, reduced bias toward the majority class. Furthermore, patient-specific data further enriched visual representations and improved diagnostic precision. These results confirm that Heart-ViT benefits from incorporating clinically meaningful variables. These findings align with previous reports linking factors such as fetal growth, the presence of a single umbilical artery, gestational age, and maternal age with CHDs risk and diagnostic detectability (21–24), reinforcing the clinical validity of the multimodal design. The fetal growth percentile is the most influential factor, underscoring the importance of fetal development metrics in identifying CHDs. The multimodal approach mirrors real-world clinical practice, where diagnostic decisions rely on both imaging and patient history.

Overall, these findings confirm that a multimodal dataset provides a strong foundation for CHD detection compared to image-only approaches. Processing imaging information through attention mechanisms and integrating clinical data via the model’s normalization layers demonstrates strong potential for improving diagnostic accuracy. Future work may explore the integration of complementary imaging modalities and longitudinal data to further refine diagnostic precision. This framework establishes a solid groundwork for developing automated diagnostic tools for prenatal CHD and underscores the potential of advanced AI methods in this domain, paving the way toward reliable and personalized early screening in prenatal care.

## Data Availability

The dataset and analytical framework will be made publicly available to facilitate further research and contribute to the improvement of early CHDs detection. The Heartbeat dataset and the source code of Heart-ViT are publicly available at: https://github.com/BCV-Uniandes/Heartbeat.git.
